# Real‐World 10‐Year Outcomes of Anti‐VEGF Therapy for Neovascular Age‐Related Macular Degeneration: A Meta‐Analysis

**DOI:** 10.1111/ceo.14559

**Published:** 2025-05-24

**Authors:** Kimberly Spooner, Geoffrey Broadhead, Samantha Fraser‐Bell, Thomas Hong, James G. Wong, Andrew A. Chang

**Affiliations:** ^1^ Cureos Research Sydney Australia; ^2^ Save Sight Institute University of Sydney Sydney Australia; ^3^ Graduate School of Health The University of Technology Sydney Australia; ^4^ Canberra Eye Surgeons Canberra Australia; ^5^ Sydney Eye Hospital Sydney Australia; ^6^ Royal North Shore Hospital St Leonards Australia; ^7^ Sydney Retina Clinic Sydney Australia; ^8^ Strathfield Retina Clinic Sydney Australia

**Keywords:** anti‐VEGF, durability, long‐term, neovascular AMD

## Abstract

**Background:**

This study examines the long‐term effectiveness of anti‐VEGF therapy in managing neovascular age‐related macular degeneration (nAMD). Despite the well‐established short‐term improvements of anti‐VEGF therapy, there is limited data on its continued efficacy over extended periods. This meta‐analysis synthesises real‐world data to evaluate anti‐VEGF therapy's long‐term outcomes systematically.

**Methods:**

We conducted a comprehensive literature review across PubMed, EMBASE and Cochrane databases, focusing on studies that reported outcomes of anti‐VEGF treatment for nAMD over a decade. The analysis included pooling baseline patient characteristics, study designs, sample sizes and changes in visual acuity (VA) over 10 years.

**Results:**

Our search produced 12 observational studies encompassing 7509 eyes, with 1274 completing 10‐years of follow‐up. The most substantial improvement in VA was observed in the first year following the initiation of anti‐VEGF therapy. On average, there was a decline of 8.11 letters in VA after 10 years from baseline (95% CI −10.83 to −5.39, *p* < 0.01). In some cases, VA reverted to baseline levels after 10 years; in others, it declined significantly below baseline. Meta‐regression showed that mean VA change was greater in those with a lower baseline VA and those treated with a higher number of injections over 10‐years(*p* < 0.01).

**Conclusion:**

Our findings suggest that the mean visual acuity of eyes treated for nAMD deteriorates progressively over the long‐term from two years after starting treatment. Regular injections appear crucial for preserving maximum vision. While our analysis did not identify an increased incidence of serious ocular adverse events, the long‐term impact of anti‐VEGF therapy on geographic atrophy remains unclear and warrants further investigation.

## 1 Introduction

Neovascular age‐related macular degeneration (nAMD) is an increasingly prevalent and progressively degenerative disease characterised by vision loss with an estimated global prevalence of 0.37% in people aged 50 and over [1]. The hallmark of nAMD is macular neovascularisation (MNV), a process of pathologic angiogenesis [2], of which vascular endothelial growth factor (VEGF) is a critical mediator [3]. Current therapeutic strategies for nAMD primarily focus on blocking the effects of VEGF with VEGF inhibitors [4]. Despite VEGF blockade, there can be progressive photoreceptor damage, atrophy, subfoveal fibrosis and ultimately irreversible vision loss [3].

The pivotal MARINA, ANCHOR and VIEW studies established the efficacy of ranibizumab and aflibercept in eyes with nAMD [5, 6], in 2‐year randomised clinical trials (RCTs) [7, 8, 9]. Extension studies have extended these observations to 5–7 years, albeit with limitations due to potential biases from high patient attrition [5].

A recent systematic review indicated a significant number of patients cease treatment with VEGF inhibitors, with approximately 50% of patients discontinuing treatment by 24 months. The onset of non‐persistence can be seen early, with a significant proportion of patients stopping treatment within the first 6–12 months [10]. The long‐term efficacy of continuous anti‐VEGF therapy in nAMD remains a significant field for further investigation.

Neovascular AMD is a chronic and progressive condition that often necessitates extended periods of anti‐VEGF treatment. Long‐term data capturing the experiences of real‐world patients undergoing continuous anti‐VEGF therapy for nAMD are providing insight into the challenges of remaining on therapy [11, 12, 13, 14]. Exploring the long‐term management strategies and the resulting clinical outcomes is critical to our understanding of the trajectory of disease outcomes. Therefore, our review examines the 10‐year outcomes reported in the literature, offering insights into the long‐term efficacy of anti‐VEGF therapy in AMD patients.

## 2 Methods

### 2.1 Search Strategy & Eligibility Criteria

This review followed the Preferred Reporting Items for Systematic Reviews and Meta‐Analysis (PRISMA) guidelines [15]. We conducted an extensive literature search in electronic databases, including PubMed, Embase and Cochrane, up to March 2023. The search utilised the following keywords and Boolean operators: (‘anti‐VEGF’ OR ‘vascular endothelial growth factor inhibitors’) AND (‘age‐related macular degeneration’ OR ‘AMD’ OR ‘neovascular AMD’ OR ‘nAMD’) AND (‘long‐term’ OR ‘outcomes’ OR ‘persistence’ OR ‘adherence’) AND (‘aflibercept’ OR ‘bevacizumab’ OR ‘ranibizumab’ OR ‘intravitreal injections’). Additionally, we manually examined the reference lists of selected articles for further relevant studies.

Two reviewers independently assessed eligibility for inclusion, with a third acting as a mediator in case of discrepancies. We included both prospective and retrospective studies that discussed anti‐VEGF treatment for nAMD for up to 10 years.

The search was focused on peer‐reviewed clinical studies published in English. Restrictions were not set on patient age, baseline visual acuity (VA), or initial anatomical measurements; however, they required a minimum follow‐up duration of 10 years. In instances of incomplete data, authors were contacted for further clarification or additional information.

### 2.2 Data Collection

Two authors independently screened and extracted data, excluding studies that did not meet the criteria and performed full‐text reviews. The data extracted included study methodology, patient details, intervention specifics and outcomes, including mean change in VA and central macular thickness (CMT) over 10 years and injection frequency.

### 2.3 Qualitative Assessment

For each included study, we produced a detailed record of the methodology employed, the inclusion and exclusion criteria, the intervention regimen, and the proportion of patients lost to follow‐up. The following baseline characteristics of the study populations were compared to ensure data homogeneity: age and gender distributions and baseline VA. The methodological quality of each study was scored using the Downs and Black checklist [16], with scores reflecting reporting of quality and external and internal validity. A third author resolved disagreements in reviews. Heterogeneity *p*‐values were calculated as indicators of potential bias across the studies.

### 2.4 Data Synthesis and Analysis

The primary measure for analysis was the difference in mean change from baseline across treatment groups for each outcome variable. Snellen acuity measurements were converted to logMAR letters for uniformity [17]. Secondary outcomes included the proportion of patients experiencing a change of 10 letters or more. Adverse events were summarised to assess safety outcomes.

The Cochrane Handbook was used to acquire standard deviation (SD) from range, median, or *p*‐value when present [18]. Different correlation coefficients were used for sensitivity analysis.

The data analysis was carried out using Comprehensive Meta‐Analysis software. After verifying data accuracy, we estimated statistical heterogeneity among studies using the I2 statistic, determining the appropriate model (fixed‐effects or random‐effects) for analysis. Continuous data, including mean changes in VA and CMT with 95% confidence intervals (CI), and dichotomous data, such as patient proportions, were analysed. Publication bias was assessed using Egger's linear regression and funnel plots.

We performed a meta‐regression analysis. Based on existing evidence, pre‐selected moderators were chosen. The moderators of interest were age at baseline, baseline VA, number of injections and treatment schedule. The output variable considered was the mean VA change in logMAR letters at 10 years.

The present study is based on previously published articles and does not imply any new studies of human participants. This work did not necessitate ethical approval as it did not include human participants or animal subjects.

## 3 Results

### 3.1 Description of Studies

**FIGURE 1:**
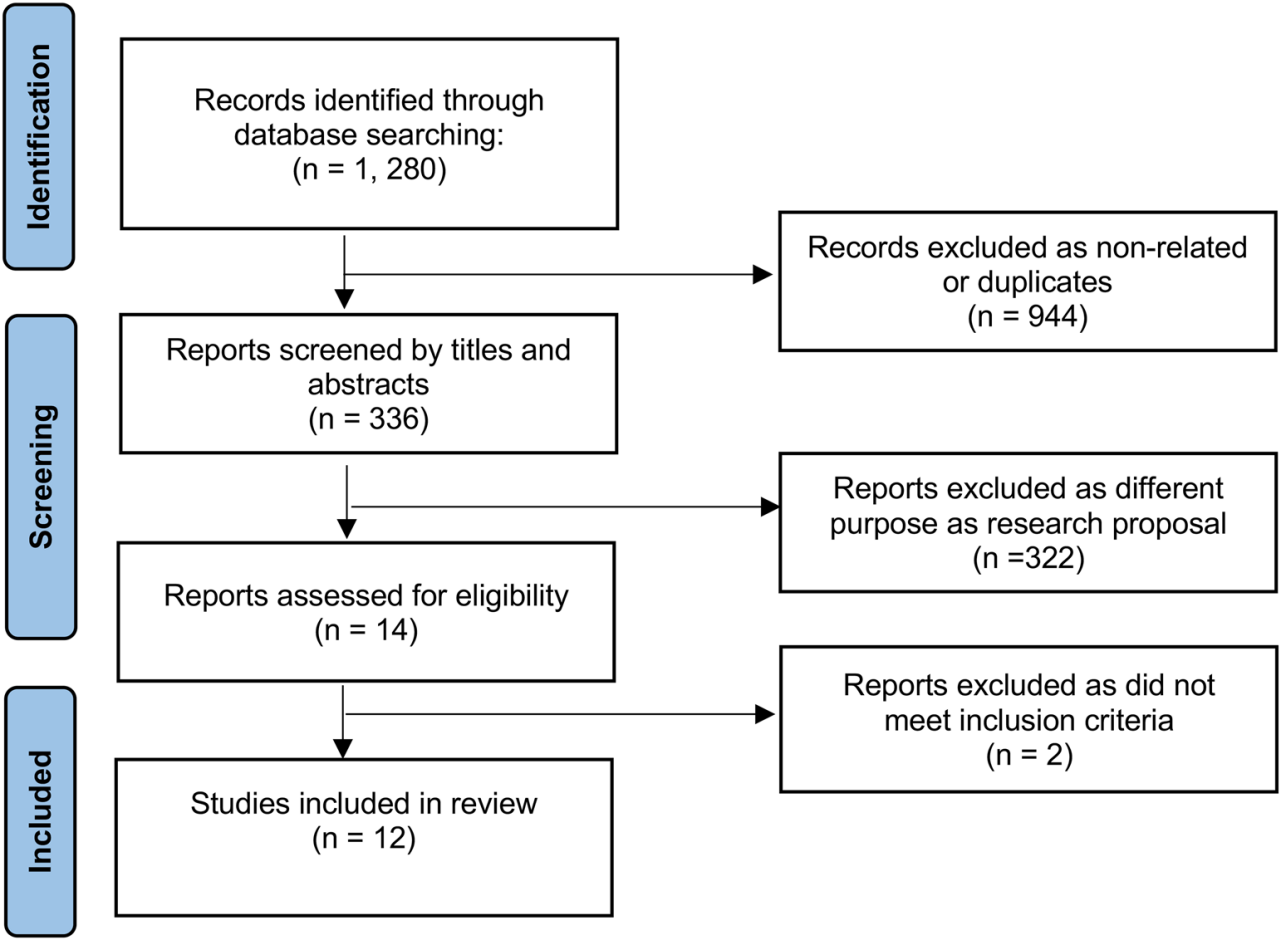
Preferred reporting items for systematic reviews and meta‐analysis (PRIMSA) flow diagram.

A total of 1280 potential articles were initially identified; 1268 non‐related and duplicate articles were excluded (Figure 1). Thus, 12 studies with a total of 7509 eyes were included in the meta‐analysis [11, 12, 13, 14, 19, 20, 21, 22, 23, 24, 25, 26]. One study reported the outcomes of two heterogeneous study groups [11], therefore, the study characteristics and statistical analyses were based on individual study populations instead of entire studies. All studies were retrospective, with two retrospectively analysing prospectively collected data [11, 14].

Information on patients lost to follow‐up (LTFU) was provided in 10 studies [11, 12, 14, 19, 21, 22, 23, 24, 25, 26].

### 3.2 STUDY CHARACTERISTICS And QUALITY

The meta‐analysis included 7509 eyes, with 1274 (17%) completing 10 years of follow‐up. Study sizes for those completing a decade ranged from 8 to 293 eyes. Geographic distribution included 10 European studies [11, 13, 14, 19, 20, 21, 22, 23, 24, 25], one in North America [12] and two in Australia [11, 26]. Participants' mean age ranged between 73 and 80 years, with the male proportions ranging from 27% to 41%. Baseline VA ranged from 50 letters (6/30 Snellen) to 62.6 logMAR letters (6/19 Snellen equivalent). Four studies reported baseline CMT outcomes [19, 20, 25, 26], averaging from 291 to 355 μm.

Baseline study characteristics are summarised in Table 1.

**TABLE 1:** Characteristics of treatment exposures included in the meta‐analysis.

Study (ref.), location	Study design	Treatment (% proportion of injections)	Treatment protocol	Inclusion/exclusion criteria
Hujanen et al. [23], Tays Eye Centre, Tampere University hospital, Finland	Retrospective single‐centre review, covering real‐world outcomes from 2008 to 2020.	1.25 mg IVB (87%), 0.5 mg IVR (2%), 2.0 mg IVA (12%)	The treatment followed a PRN regimen, with an initial dosing of three monthly injections, followed by monitoring and additional injections if required.	Main inclusion criteria: Treatment‐naïve eyes diagnosed with nAMD. Eyes with ICD‐10 code H35.31 for nAMD and at least one intravitreal injection between 2008 and 2020. Main exclusion criteria: Eyes that began treatment before 2008. Eyes treated with laser photocoagulation, photodynamic therapy, or intravitreal steroids. Eyes without baseline visual acuity (VA) data or those treated for conditions other than nAMD.
Young et al. [22], Edinburgh, Scotland	Single‐centre, retrospective cohort study conducted at a public tertiary referral hospital.	0.5 mg IVR, 2.0 mg IVA	Initial loading dose of three monthly injections followed by a PRN regimen. Those requiring continued treatment after 2013 were offered a switch to aflibercept.	Main inclusion criteria: Patients who began receiving intravitreal anti‐VEGF treatment for nAMD between 2007 and 2008, with at least 10 years of follow‐up. Main exclusion criteria: Patients receiving intravitreal injections for other macular conditions unrelated to AMD.
Upasani et al. [24], Mid Yorkshire NHS Trust, Wakefield, United Kingdom	Single‐centre, retrospective cohort study, assessing 10‐year outcomes from 2009 to 2019.	0.5 mg IVR, 2.0 mg IVA	All patients received an initial loading dose of three monthly injections of ranibizumab, followed by PRN. Patients who switched to aflibercept received three loading doses followed by fixed bimonthly dosing for the first year after the switch.	Main inclusion criteria: Treatment‐naïve eyes diagnosed with nAMD initiated on anti‐VEGF therapy between January and December 2009. Main exclusion criteria: Eyes that were previously treated with other therapies for nAMD, such as laser photocoagulation, photodynamic therapy, or intravitreal steroids.
Cheema et al. [25], Royal Victoria Infirmary, Newcastle upon Tyne, United Kingdom	Retrospective, single‐centre cohort study.	0.5 mg IVR, 2.0 mg IVA	Patients received an induction phase of three monthly injections of ranibizumab followed by PRN. After 2013, patients could transition to aflibercept, with most of them switching in an effort to reduce treatment burden.	Main inclusion criteria: Treatment‐naïve eyes diagnosed with nAMD, starting anti‐VEGF treatment between January 2007 and December 2009. Main exclusion criteria: Eyes previously treated for nAMD with other therapies (e.g., laser photocoagulation, photodynamic therapy) were excluded.
Brynskov et al. [14], Zealand University Hospital, Roskilde, Denmark	Retrospective single‐centre cohort study, reviewing real‐world outcomes from 2007 to 2019.	0.5 mg IVR, 2.0 mg IVA	The treatment protocol followed a PRN regimen. After 2015, the follow‐up period was extended to 8 weeks with aflibercept as part of national treatment guidelines.	Main inclusion criteria: Treatment‐naïve eyes diagnosed with nAMD that received at least one intravitreal injection between January 2007 and January 2019. Main exclusion criteria: Eyes with no follow‐up visit or no baseline VA data, and those where the diagnosis of nAMD was redrawn.
Chandra et al. [13], Moorfields Eye Hospital, London, United Kingdom	Retrospective, single centre cohort study.	0.5 mg IVR, 2.0 mg IVA	The treatment followed a PRN regimen from 2008 to 2013. From 2013 onwards, patients were switched to aflibercept, with a TAE protocol introduced later, after the loading phase.	Main inclusion criteria: Patients diagnosed with nAMD who began anti‐VEGF therapy between 2008 and 2009 and had at least 10 years of follow‐up. Main exclusion criteria: Eyes that were not treatment‐naïve or those that did not complete the required follow‐up period.
Spooner et al. [26], Sydney Retina Clinic, Sydney, Australia	Retrospective, single centre cohort study.	0.5 mg IVR, 2.0 mg IVA	The treatment followed a TAE regimen after initial loading phase of three monthly injections of ranibizumab. The decision to switch to aflibercept was based on the physician's discretions, depending on patient's response to ranibizumab.	Main inclusion criteria: Anti‐VEGF naïve eyes diagnosed with nAMD that commenced treatment between November 2006 and December 2009. Main exclusion criteria: Eyes previously treated with anti‐VEGF therapy, eyes with other macular diseases (e.g., diabetic retinopathy or retinal vein occlusion), or eyes that received fewer than three injections in the first year.
Wolff et al. [20], Maison Rouge Ophthalmologic Centre, Strasbourg, France	Retrospective, single centre cohort study.	0.5 mg IVR, 2.0 mg IVA	Patients followed a PRN regimen.	Main inclusion criteria: Eyes with nAMD that initiated anti‐VEGF treatment between January 2006 and April 2008 and were tracked in the Fight Retinal Blindness (FRB!) registry for 10 years. Main exclusion criteria: Eyes with less than 10 years of treatment were excluded.
Gerding et al. [21], Switzerland	Retrospective, institutional case series analysis.	0.5 mg IVR, 2.0 mg IVA	All patients received three monthly loading doses of ranibizumab and were examined during the first year. After the first year, patients followed an individualised PRN regimen.	Main inclusion criteria: Only eyes that met the inclusion criteria or the MARINA/ANCHOR studies were included. Main exclusion criteria: Exclusion criteria are not explicitly mentioned, however, the selection was based on MARINA/ANCHOR study criteria.
Gillies et al. [11], Australia, New Zealand & Switzerland	Multi‐centre, retrospective, comparative, interventional case series	1.25 mg IVB, 0.5 mg IVR, 2.0 mg IVA	Treatment followed a TAE regimen in Australia and New Zealand, while a PRN regimen was more commonly followed in Switzerland.	Main inclusion criteria: Treatment‐naïve eyes diagnosed with nAMD, starting treatment between January 1, 2006, and December 31, 2008, with follow‐up available through 2019. Main exclusion criteria: Eyes that did not complete 10 years of continuous treatment were excluded from the main outcomes analysis but included in some secondary analyses.
Starr et al. [12], United States	Single‐centre, retrospective, cohort study of prospectively collected data.	0.5 mg IVR, 2.0 mg IVA	Treatment followed a PRN regimen after an initial loading dose of three monthly injections.	Main inclusion criteria: Patients with treatment‐naïve eyes diagnosed with nAMD who initiated anti‐VEGF therapy between 2007 and 2009. Main exclusion criteria: Patients with other macular diseases or eyes treated for nAMD prior to the study period.
Garweg et al. [19], Berner Augenklinik, Switzerland	Retrospective, single‐centre cohort study	0.5 mg IVR, 2.0 mg IVA	Treatment followed a PRN regimen, following at lleast three loading injections of ranibizumab.	Main inclusion criteria: Patients diagnosed with CNV due to nAMD, treated with intravitreal injections between 2007 and 2012, and followed for at least 4 years. Main exclusion criteria: Excluded patients with other causes of CNV, those with diabetic retinopathy, uveitis, or other vascular ocular diseases, and those who did not attend scheduled follow‐up visits.

Abbreviations: CNV, choroidal neovascularisation; FRB!, fight retinal blindness registry; IVA, intravitreal aflibercept (Eylea); IVB, intravitreal bevacizumab (Avastin); IVR, intravitreal ranibizumab (Lucentis); nAMD, neovascular age‐related macular degeneration; OCT, optical coherence tomography; PRN, pro re nata (as needed after three initial monthly loading doses); TAE, treat and extend.

Anti‐VEGF treatment regimens in the included studies primarily followed two approaches: pro re nata (PRN) and Treat‐and‐Extend (TAE). PRN, meaning ‘as needed’, involves close monitoring with injections administered in response to disease activity, while TAE regimens use proactive scheduling with extended intervals between injections based on disease stability. These regimens were variably employed across studies, either alone or in transition over time, with the most common treatment regimen being *pro re nata* (PRN). Detailed information about the follow‐up and treatment regimen is summarised in Table 2.

**TABLE 2:** Baseline characteristics of included studies.

Study (ref.)	Location	Treatment (% proportion of injections)	Eyes at baseline (*n*)	Eyes with 10‐years (*n*)	Age (SD)	Baseline BCVA (SD) LOGMAR letters [Snellen]	Baseline CFT (SD) μm	Patients LTFU
Starr et al. [12]	USA	0.5 mg IVR, 2.0 mg IVA	354	130	74.4 (7.7)	54.5 (6.9) [6/24 Snellen]	291.0 (94.0)	Reasons for loss to follow‐up were not explicitly detailed in the manuscript.
Garweg et al. [19]	Berner Augenklinik, Switzerland	0.5 mg IVR, 2.0 mg IVA	104	8	77.3 (6.9)	59.0 [6/19 Snellen]	608	By the end of the study, 16 patients had died, 29 eyes (27.9%) had discontinued treatment for unknown reasons, and 53 eyes (19.8%) had returned to their local ophthalmologists for further therapy.
Gillies et al. [11]	Australia & New Zealand	NR	474	132	79.1 (7.7)	60.7 (17.0) [6/19 Snellen]	NR	Reasons for treatment discontinuation were reported for 477 eyes (76%). The main reasons included: Patient transferred to another doctor (30%) Death (14%) Treatment considered futile (20%) Patient declined further treatment (9%)
Gillies et al. [11]	Switzerland	NR	321	37	79.3 (6.9)	61.6 (14.0) [6/19 Snellen]	NR	
Gerding et al. [21]	Switzerland	0.5 mg IVR, 2.0 mg IVA	104	30 (29% of initial cohort)	77.6 (7.3)	50 (14.4) [6/30 Snellen]	NR	Not specifically mentioned, but by year 10, 29% of eyes remained in follow‐up.
Brynskov et al. [14]	Zealand University Hospital, Roskilde, Denmark	0.5 mg IVR, 2.0 mg IVA	4678	74 (approximately 1.6% of the initial cohort)	72.9 (7.0)	61.5 (12.1) [6/19 Snellen]	NR	55% (2566 eyes) were discontinued due to a variety of reasons, including: Death Disease inactivation Intractable disease Patients unwilling or unable to proceed with treatment General illness or relocation.
Chandra et al. [13]	Moorfields Eye Hospital, London, United Kingdom	0.5 mg IVR, 2.0 mg IVA	611	149	74.5 (7.8)	59.5 (13.1) [6/19 Snellen]	298.7 (87.9)	No patients were lost to follow‐up as the analysis included only those who completed 10 years of treatment. However, some patients were switched to aflibercept during the study period.
Spooner et al. [26]	Sydney Retina Clinic, Sydney, Australia	0.5 mg IVR, 2.0 mg IVA	1046	293 (28% of initial cohort)	74.5 (8.9)	56.6 (16.3) [6/24 Snellen]	355.5 (107.8)	Of the 753 eyes that did not complete 10 years of follow‐up: 55% (417 eyes) were lost due to death. 38% (288 eyes) transferred to another specialist closer to home. 2% (14 eyes) discontinued treatment due to futility. 14% (107 eyes) had unknown reasons for discontinuation
Wolff et al. [20]	Maison Rouge Ophthalmologic Centre, Strasbourg, France	0.5 mg IVR, 2.0 mg IVA	116	116	76.0 (6.1)	57.5 (17.5) [6/24 Snellen]	NR	No specific reasons for loss to follow‐up were mentioned, as the analysis focused on eyes with complete 10‐year follow‐up.
Upasani et al. [24]	Mid Yorkshire NHS Trust, Wakefield, United Kingdom	0.5 mg IVR, 2.0 mg IVA	223	60 (27% of initial cohort)	75.9 (7.1)	51.0 (16.1) [6/24 Snellen]	339.1 (125.5)	Of the 223 eyes, 163 eyes (73%) did not complete the 10‐year follow‐up. The main reasons for discontinuation were: Death (47.2%) Permanent foveal damage (21.5%) Patients discharged as clinically stable (12.9%) Patient refusal to attend or transfer of care to another hospital (8%) No reason recorded (10.4%)
Cheema et al. [25]	Royal Victoria Infirmary, Newcastle upon Tyne, United Kingdom	0.5 mg IVR, 2.0 mg IVA	197	90 (45.9% of initial cohort)	79.2 (8)	62.6 (13.6) [6/19 Snellen]	NR	51.4% of patients were lost to follow‐up due to death. Other reasons included macular atrophy and fibrosis, poor general health, difficulties with transportation, or shared decision‐making about discontinuing treatment based on disease progression
Young et al. [22]	Edinburgh, Scotland	0.5 mg IVR, 2.0 mg IVA	216	112	79.1 (6.9)	59.6 (14.5) [6/19 Snellen]	NR	Mortality was the primary reason for loss to follow‐up, with 52.6% (113/216) of patients deceased by the 10‐year follow‐up point. The study also mentions that some records were not included for visual acuity analysis due to destruction of paper medical records after patient death.
Hujanen et al. [23]	Tays Eye Centre, Tampere University hospital, Finland	1.25 mg IVB (87%), 0.5 mg IVR (2%), 2.0 mg IVA (12%)	3844	43	80 (8.15)	55 (20) [6/24 Snellen]	NR	26% were lost to follow‐up by the end of year 1, 47% by year 2, and 92% by year 10. Reasons for LTFU include poor treatment outcomes (44%), disease inactivation (26%), poor general health (10%), other or unknown reasons (9%), patient refusal of treatment (7%), and death (5%)

Abbreviations: BCVA, best corrected visual acuity; CFT, central foveal thickness; IVA, Intravitreal aflibercept (Eylea); IVB, intravitreal bevacizumab (Avastin); IVR, intravitreal ranibizumab (Lucentis); LTFU, lost to follow‐up; *n*, number; NR, not reported; SD, standard deviation.

The number of participants lost and reasons for their withdrawal were provided in seven included reports. Rates of participants lost to follow‐up were reported between 54% and 92% after 10 years of therapy. The possibility of bias could not be excluded because explanations of the imbalanced distributions were not always provided. Although several unclear risks of bias were present, all studies were graded as moderate quality.

### 3.3 Visual Acuity Outcomes

**FIGURE 2:**
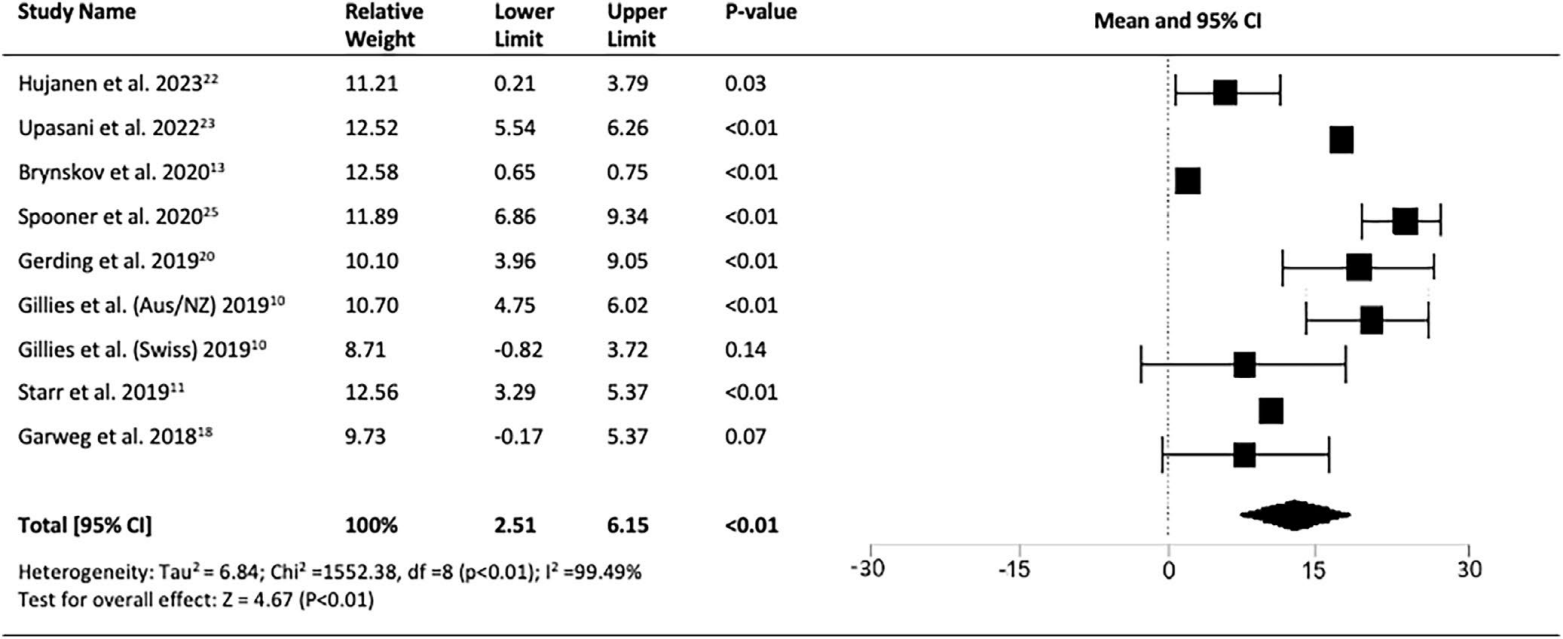
Pooled change in VA at 1‐year (LogMAR) letters.

In eight of the twelve studies, VA was assessed after one year (*n* = 807 eyes). The improvement in VA after 1 year of treatment was rapid; however, a random‐effects model was used due to the significant heterogeneity. All eight studies demonstrated a post‐treatment gain in VA relative to baseline, with a considerable difference emerging in four studies. The mean improvement in VA at 1 year was 4.3 letters (95% CI = 2.51 to 6.15, *p* < 0.01) (Figure 2).

After 10 years of treatment, regression of difference in means on regimen was statistically significant, favouring a TAE regimen over a PRN regimen. A TAE regimen showed a coefficient of +3.72 LogMAR letters over PRN (95% CI = 0.91–5.1, *p* = < 0.01).

**FIGURE 3:**
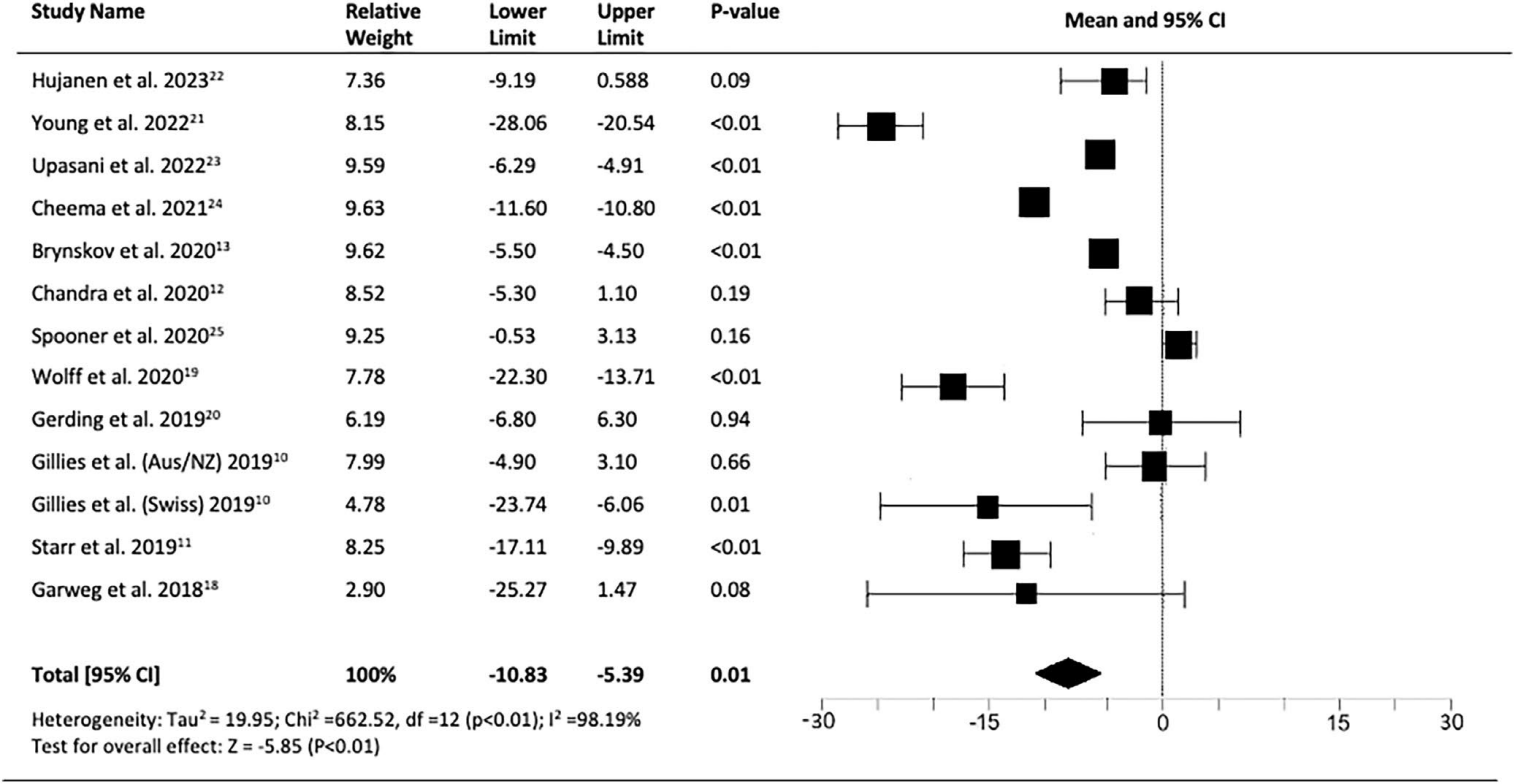
Pooled change in visual acuity (VA) at 10‐years (LogMAR) letters.

The mean change in VA after 10 years of anti‐VEGF treatment was a loss of 8.11 letters (95% CI = −10.83 to −5.39, *p* < 0.01), resulting in a final VA ranging from 39.9 to 60.1 letters (Snellen equivalent of 20/160 to 20/80) (Figure 3).

**FIGURE 4:**
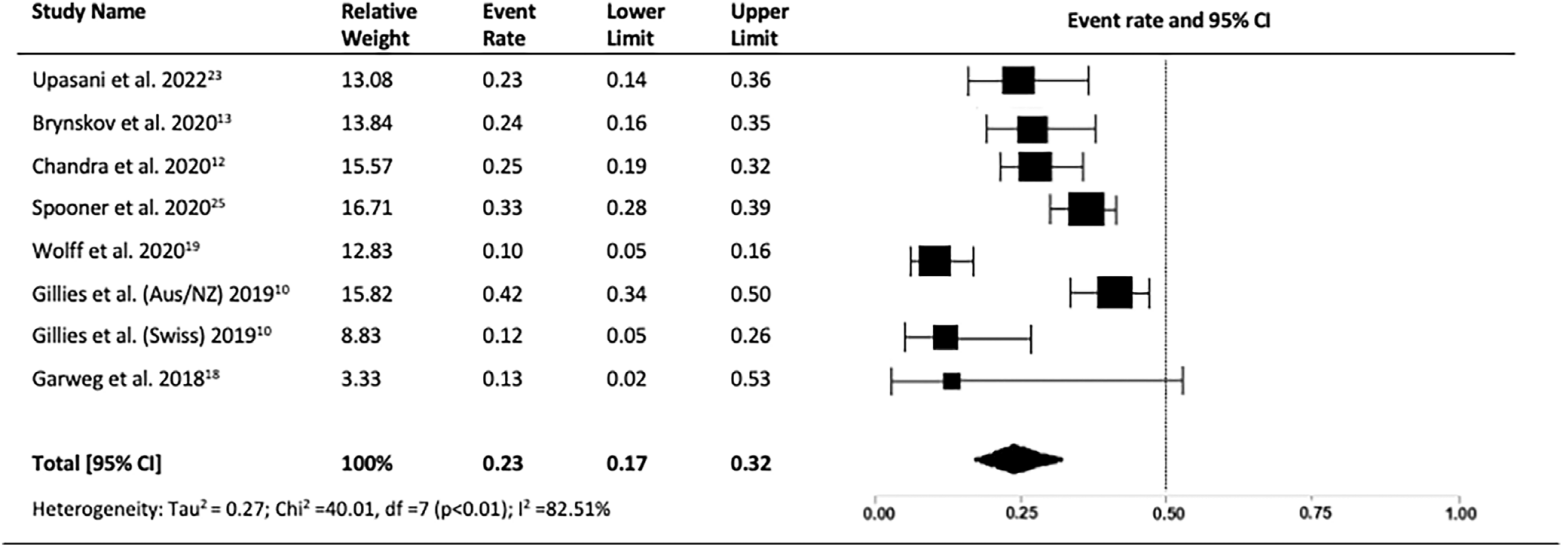
Proportion eyes gaining > 10 letters at year 10.

**FIGURE 5:**
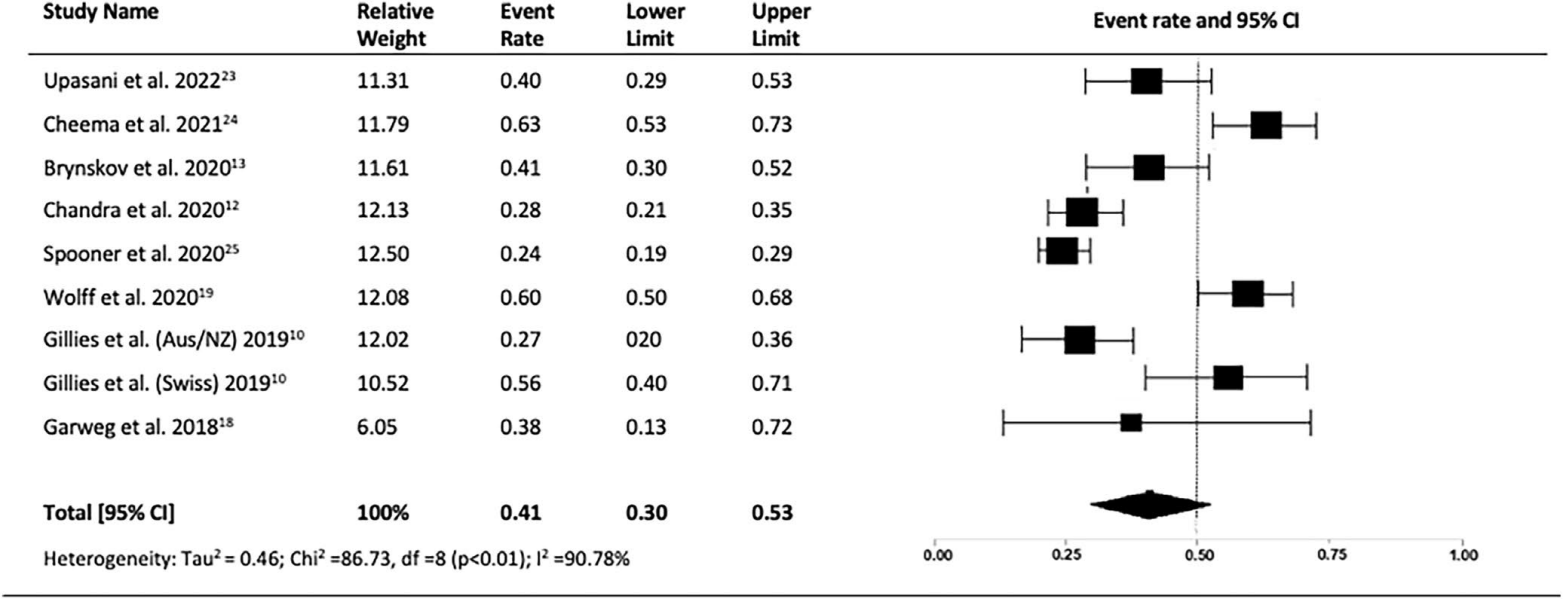
Proportion eyes losing > 10 letters at year 10.

Seven and eight studies reported the proportions of eyes gaining and losing 10 or more LogMAR letters at 10 years, respectively. The proportion of eyes gaining 10 or more letters after 10 years of treatment was 23% (95% CI = 0.17–0.32, *p* < 0.01, I2 = 82.51%) (Figure 4). A significant proportion of eyes lost 10 or more letters: 41% (95% CI = 0.30–0.53, *p* < 0.01, I2 = 90.78%) (Figure 5).

**FIGURE 6:**
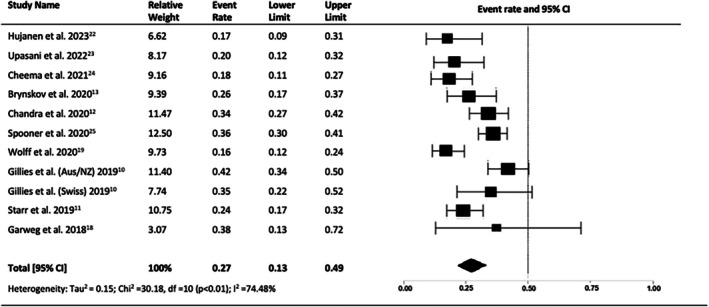
Proportion eyes achieving a final visual acuity of > 70 letters at year 10.

**FIGURE 7:**
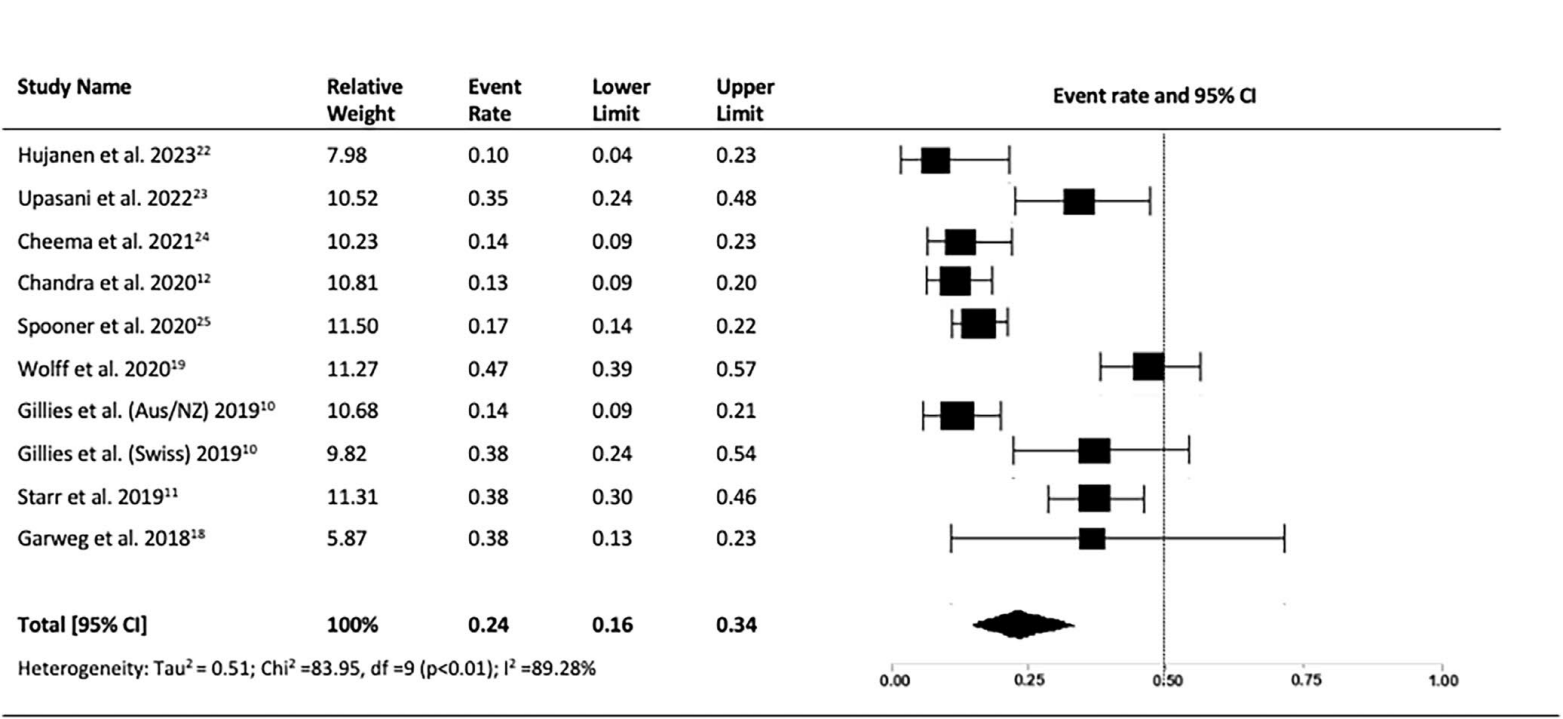
Proportion eyes achieving a final visual acuity of < 35 letters at year 10.

The proportion of patients achieving a final VA of ≥ 70 letters (Snellen equivalent 6/12) was 27% (95% CI = 0.22–0.33, *p* < 0.01, I2 = 74.48%) (Figure 6), and the proportion of patients with a final vision of ≤ 35 letters (Snellen equivalent ≤ 6/60) was 24% (95% CI = 0.16–0.34, *p* < 0.01, I2 = 89.28%) (Figure 7).

### 3.4 Anatomical Outcomes

**FIGURE 8:**
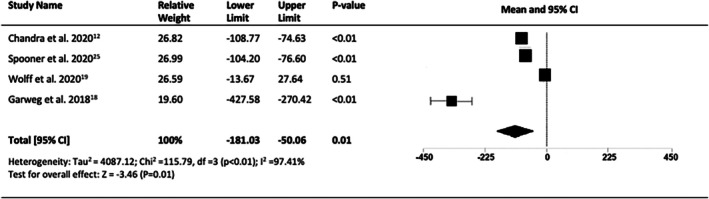
Change in central macular thickness (CMT) at 10‐years.

**FIGURE 9:**
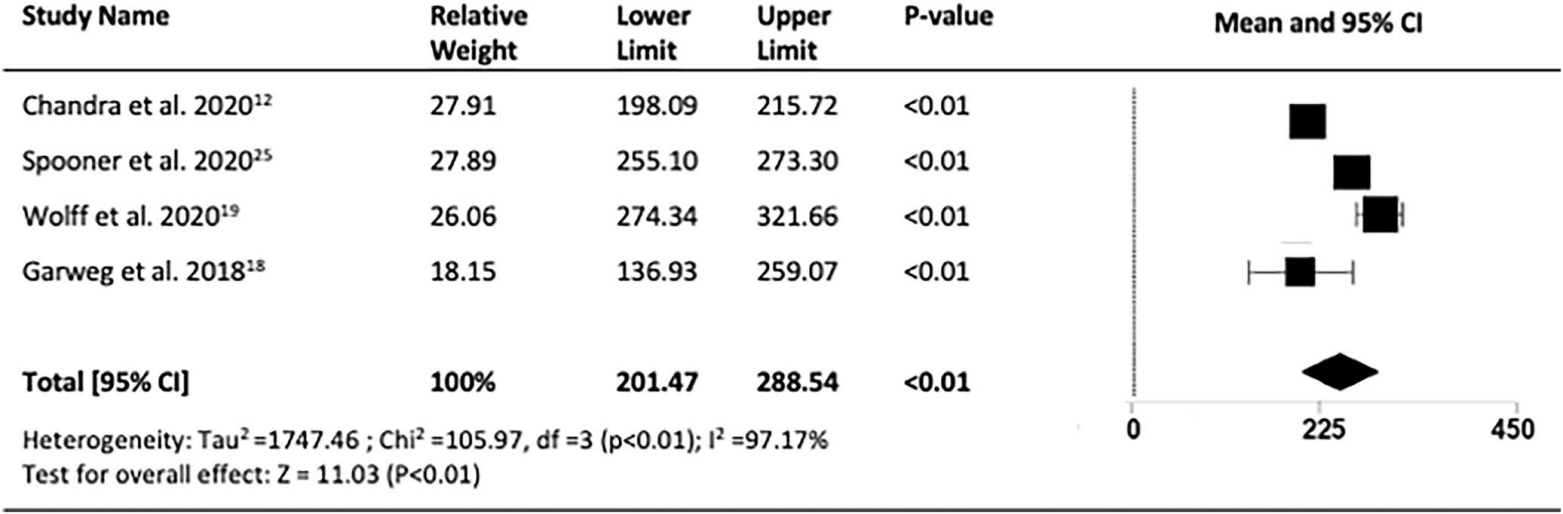
Final central macular thickness (CMT) at 10 years.

Four of the 12 studies (*n* = 566 eyes) reported changes in CMT. CMT was determined based on SD‐OCT in two studies and not described in 2. All studies reported statistically significant reductions in macular thickness, with an overall decrease at 10 years of 115.54 μm (95% CI = −181.03 to −50.06, *p* < 0.001) (Figure 8) for a final mean CMT (five studies, *n* = 656) of 265.2 μm (95% CI = 214.55 to 315.85, *p* < 0.001, I2 = %) (Figure 9).

**FIGURE 10:**
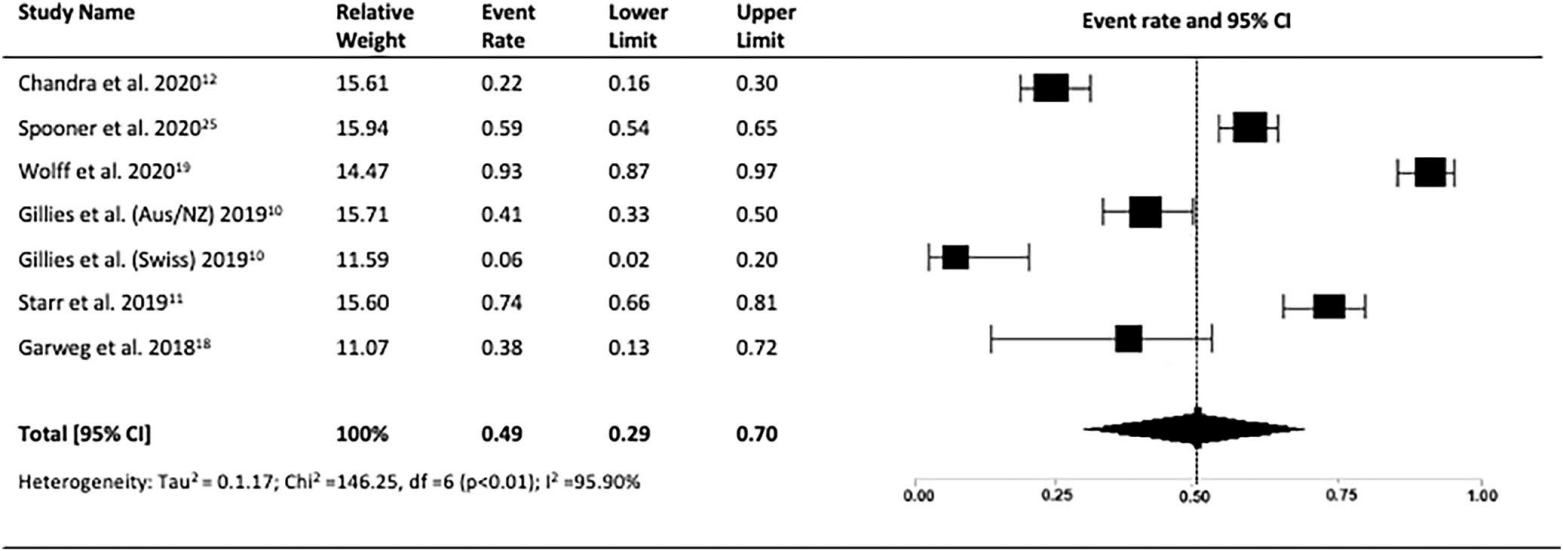
Proportion of patients with macular atrophy at 10 years.

The presence of macular atrophy was determined in most of the studies by recorded clinical examination or OCT findings. Seven studies (*n* = 865 eyes) reported the proportion of patients having macular atrophy after 10 years of anti‐VEGF therapy at 49% (95% CI = 0.29 to 0.67, *p* = 0.19, I2 = 95.90%) (Figure 10).

### 3.5 Number of Injections

Treatment frequency varied across studies due to differing regimens; therefore, a random‐effects model was utilised. Most studies employed a labelled regimen of three monthly loading doses. Following this, nine studies adopted a PRN approach after loading doses had been given. Two studies followed a treat and extend regimen, and the remaining followed a combination of PRN/TAE regimens.

**FIGURE 11:**
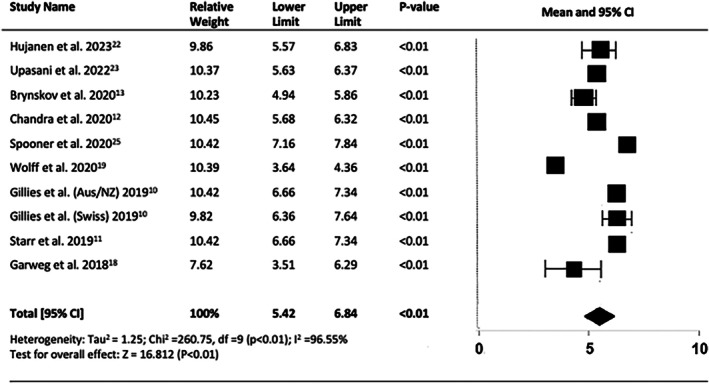
Number of injections administered in year 1.

The mean treatment frequency of injections in all available studies was highest in the first year, 6.13 (95% CI = 5.42–6.84, *p* < 0.01) (Figure 11), followed by relatively consistent frequency values between a mean of 4.1 and 5.1 injections per year.

**FIGURE 12:**
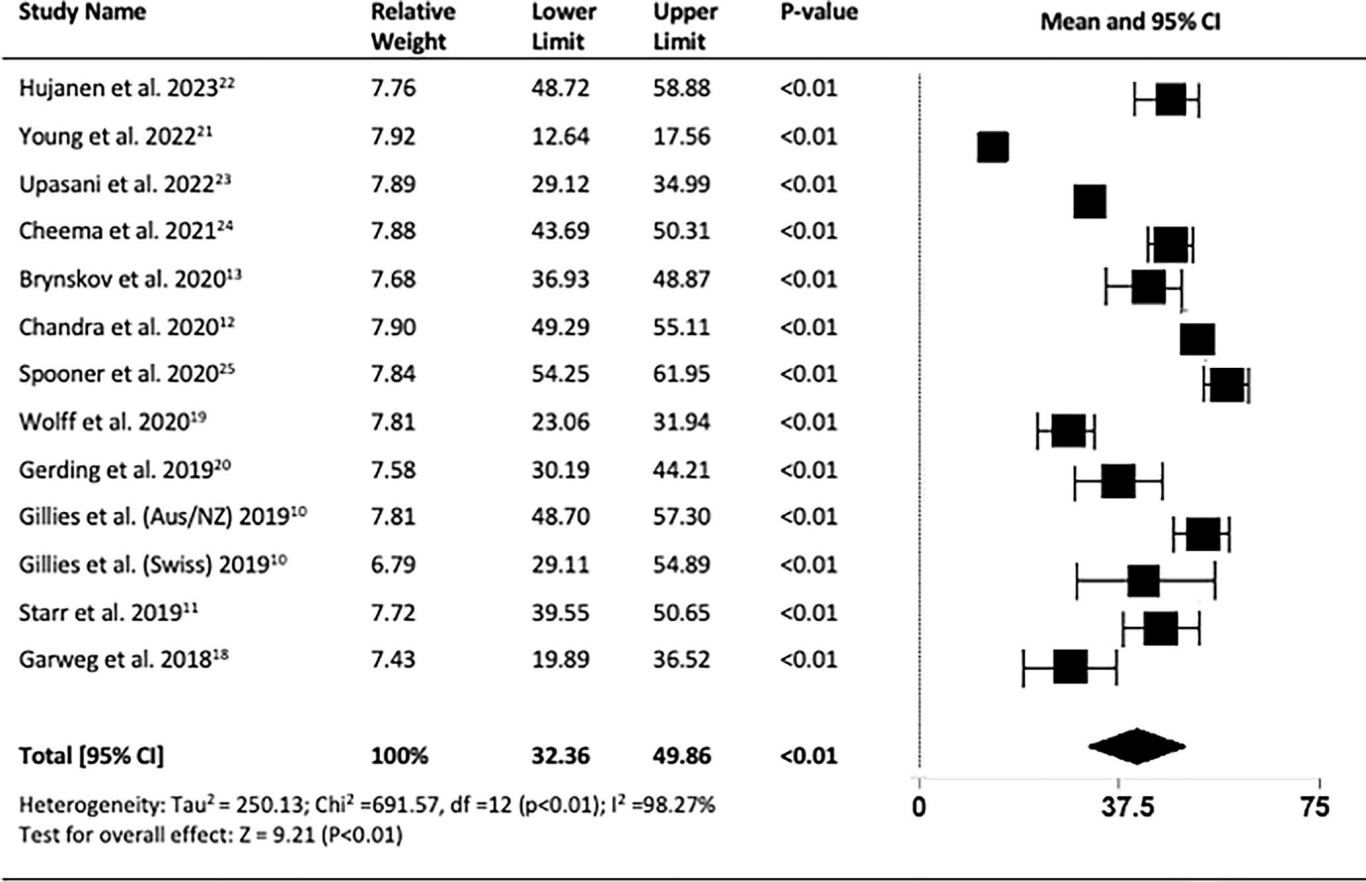
Cumulative total number of injections administered over 10 years.

At 10 years, the combined number of anti‐VEGF injections administered was 41.11 (95% CI = 32.36 to 49.86, *p* < 0.001) (Figure 12).

Ocular serious adverse events (SAEs) were rare, with endophthalmitis rates ranging from 0.007% to 0.04% per injection.

### 3.6 Sensitivity Analyses and Publication Bias

To evaluate the impact of individual studies on the overall weighted mean difference (WMD), we conducted sensitivity analyses by sequentially excluding each study. These analyses revealed that no single study disproportionately influenced the overall WMD, supporting the robustness of our conclusions.

Methodological quality assessments for five key outcomes, including changes in VA, CMT, the percentage of eyes gaining or losing 10 or more letters, the proportion of eyes achieving more than 70 or less than 35 letters, and the total number of injections, were conducted using the Downs and Black checklist. The quality of evidence for all outcome data was rated as fair to good. Notably, the most significant variance across studies pertained to the reporting category, particularly in documenting adverse events and characteristics of patients lost to follow‐up. Moderate heterogeneity was evident in the overall amalgamation of studies (*p* < 0.001). Tests for publication bias, specifically Begg's test (*p* = 0.53, continuity corrected) and Egger's test (*p* = 0.56), indicated no significant influence of publication bias on our results.

### 3.7 Meta‐Regression

A meta‐regression of difference in means analysis of the mean number of injections was statistically significant (coefficient + 0.46 LogMAR letters; 95% CI = +0.19, +0.74, *p* < 0.01). Meta‐regression demonstrated a clear trend towards better visual outcomes with higher cumulative injection numbers. For each additional injection received over 10 years, the patient would expect to gain 0.46 letters.

Conversely, a statistically significant negative correlation was observed between patients' age and effect size, indicating that older age was associated with a reduced response to treatment (coefficient + 0.54 LogMAR letters; 95% CI = −2.06–+0.99, *p* = 0.05). The regression data further highlighted a gradual decline in initial VA gains over time.

## 4 Discussion

This meta‐analysis examines the long‐term efficacy of anti‐VEGF therapy in treating nAMD. The analysis included twelve clinical studies, predominantly of small to medium size, with a moderate quality of evidence overall. Despite over 15 years of anti‐VEGF use in nAMD management, long‐term (10‐year) study data remain limited.

All studies demonstrated a favourable response to anti‐VEGF therapy, with a prompt and robust response seen within the first year of treatment. In those studies that reported anatomical outcomes, CMT reduction was significant and maintained by regular injections. Coincident with the resolution of fluid, VA improved rapidly and significantly in the first year of treatment. However, in all studies, improvement in vision was not maintained despite ongoing injections throughout the follow‐up periods. There was a clear trend of slow loss in visual gains to below the baseline. Macular atrophy (MA) was a significant cause of poor visual outcomes [27, 28, 29, 30] and was predominant in almost half of all eyes after 10 years of treatment, and may be a substantial cause of the vision loss reported. Although our analysis suggests that MA may contribute significantly to vision decline, the causal relationship between long‐term VEGF suppression and the development of MA remains uncertain. Further prospective studies are needed to delineate whether prolonged anti‐VEGF therapy accelerates atrophic changes.

The drop‐out rate of patients over time within the cited studies was high [31, 32]. It is important to note that natural mortality likely accounts for a substantial proportion of patients lost to follow‐up. Based on actuarial data, approximately 50% of individuals aged 80 years are expected to die within 10 years [33], aligning with the reported mortality rates in some studies, such as Young et al. (52.6%) and Upasani et al. (47.2%) [22, 24]. However, other studies reported considerably lower rates, such as Gillies et al. (14%) and Hujanen et al. (5%) [11, 23], possibly reflecting differing definitions or documentation practices for loss to follow‐up. This variability introduces potential bias in interpreting long‐term visual outcomes, as death with preserved vision could be considered a treatment success, in contrast to loss to follow‐up due to vision deterioration. In recent research by Finger et al., 82% of nAMD patients (*n* = 2629; 95% CI, 2590–2660) had dropped out 11 years after diagnosis [32]. The high proportion of non‐adherent and non‐persistent patients with intravitreal injection therapy is a significant limitation of this therapeutic option under real‐life conditions and may contribute to inferior results in vision [34]. Patients only completed an average of 3.7 years of regularly scheduled injections before dropping out of anti‐VEGF therapy altogether [35]. Studies such as AURA demonstrated that anti‐VEGF allows for maintaining good vision; however, lower treatment frequencies led to decreased vision over time [36], with an average of 7.9 injections needed for a 15‐letter improvement in VA at 2 years, underscoring the challenges of maintaining long‐term treatment adherence.

Analysis of the employed treatment frequency in the available long‐term literature indicates a wide range of proactive and reactive variations. This analysis compared two main treatment protocols for AMD: Treat‐and‐Extend (TAE) and Pro Re Nata (PRN). Studies in Australia and New Zealand, such as Gillies et al. [11], used the TAE regimen, while others, like Garweg et al. [19], initially followed a PRN approach. Over time, many studies transitioned from PRN to TAE, especially with the introduction of aflibercept, which allowed for longer treatment intervals and reduced burden. The shift to aflibercept, from drugs like ranibizumab, improved visual stability and reduced injection frequency, leading to better long‐term outcomes in studies such as Chandra et al. [13] and Gillies et al. [11]. The mean number of injections in all studies indicates a moderately continuous treatment frequency over time. Quantitatively, the employed treatment frequency was slightly lower than those reported in the first year of treatment (6.0 injections). Increasing research indicates that the treatment approach and the frequency of injections play a crucial role in the visual outcomes for patients with nAMD. Our study's findings support this view; there was a statistically significant link between the frequency of anti‐VEGF treatments and changes in VA. Over 10 years, we observed that each additional treatment administration is associated with an improvement of 0.46 letters in VA.

Furthermore, our findings focused on determining the efficacy of differing treatment protocols in achieving optimal results. The meta‐regression analysis revealed that proactive treatment strategies (Treat‐and‐Extend) return better results than reactive strategies (such as Pro Re Nata or PRN). This finding is further evident in real‐world studies, where meta‐analysis has shown a visual benefit to TAE regimes compared to PRN regimes [37]. In practice, several factors can impact the success of PRN treatment approaches, including issues related to management and logistics. For example, challenges like inadequate scheduling of treatment and follow‐up appointments can significantly affect treatment outcomes in real‐world scenarios. Additionally, the difficulty in consistently applying strict criteria for retreatment can lead to less‐than‐ideal outcomes for patients.

The progression of change in VA between year one and the end of year 10 of therapy can be characterised by a stabilisation of visual function with a slow long‐term trend of gradual loss of previous gains. Over time, the average increase in VA of 4.4 letters at 1 year was lost, and after 10 years, vision had deteriorated to around 1.5 lines below the baseline. These findings need to be considered in light of the natural history of untreated eyes with nAMD. Untreated eyes lose, on average, approximately 61.22 letters after 5 years and 65.93 letters after 10 years of active nAMD [38]. However, even with repeated injections, the mean vision at baseline was not sustained. Recurrence of fluid, subretinal fibrosis formation, and atrophy development are critical contributors to this.

This study has several limitations; firstly, it is important to note that throughout the extended follow‐up period, a significant number of patients were lost to follow‐up across all the included studies. While the pooled data provides a more robust analysis than individual studies and allows for a broader assessment, incomplete data across all studies limited the depth of our meta‐analyses. Additionally, during the period from which the studies were drawn, improvements in treatment regimens and medications may have had an impact on the generalisability of the findings. The presence of macula atrophy was also not documented using standardised criteria across these studies.

Another notable limitation is the lack of diversity in the study populations, particularly a paucity of patients of Asian and African descent.

The body of evidence points to a fluctuating course in the long‐term management of nAMD with anti‐VEGF therapy, marked by a gradual decline in VA below baseline levels. Regular and consistent dosing appears crucial for maintaining VA, suggesting that treatment adherence could significantly influence the long‐term outcomes in nAMD. Ongoing anti‐VEGF therapy, even over prolonged periods such as 10 years, is still beneficial in preventing maximal vision loss due to nAMD.

## Conflicts of Interest

Dr. Andrew A. Chang has consulted for Novartis, Bayer, Roche, Opthea, Apellis, Astellas, Zeiss and Alcon. Dr. Samantha Fraser‐Bell has consulted for Bayer, Novartis, Roche and Allergan an AbbVie company. Dr. James G. Wong has acted as a consultant for Bayer, Novartis, Roche and Allergan an AbbVie company. Kimberly Spooner is currently an employee of Boehringer‐Ingelheim, Germany. BI had no affiliation with this work. Thomas Hong has consulted for Bayer.

## Data Availability

Data sharing is not applicable to this article as no new data were created or analyzed in this study.
